# Evaluation of outcome indicators in trials for atopic dermatitis

**DOI:** 10.1186/s43556-025-00273-8

**Published:** 2025-05-26

**Authors:** Jingru Tian, Liqing Shi, Shuntong Kang, Dingyao Zhang, Yaqing Huang, Ming Zhao, Xu Yao, Qianjin Lu

**Affiliations:** 1 Hospital for Skin Diseases, Institute of Dermatology, Chinese Academy of Medical Sciences and Peking Union Medical College, 12 Jiangwangmiao Street, Xuanwu, Nanjing, Jiangsu 210042 China; 2 Key Laboratory of Basic and Translational Research On Immune-Mediated Skin Diseases, Chinese Academy of Medical Sciences, Nanjing, China; 3 Department of Dermatology, Hunan Key Laboratory of Medical Epigenomics, The Second Xiangya Hospital, Central South University, Changsha, Hunan China; 4 Graduate Program in Biological and Biomedical Sciences, Yale University, New Haven, CT 06510 USA; 5 Department of Pathology, Yale University, New Haven, CT 06520 USA

Dear Editor,

Atopic dermatitis (AD) is a chronic inflammatory skin condition characterized by intense itching, recurring eczematous lesions, and impaired skin barrier function [1, 2]. Over recent years, therapeutic approaches for AD have advanced significantly, with an increasing number of novel treatments entering clinical trials. These trials are crucial for validating the efficacy of new therapies, making the selection of appropriate assessment indicators more important than ever [3]. However, this remains a challenging task, as there are currently no established guidelines or standardized references for selecting outcome measures in AD randomized controlled trials (RCTs) [4]. In our study, we used a Bayesian hierarchical linear mixed model to evaluate the relative discriminative power of various outcome measures. By examining their sensitivity under different conditions, we emphasize the need for a multidimensional approach to assess trial validity, thereby providing a foundation for selecting primary outcomes in AD RCTs.

A total of 133 RCTs were included in our study (Fig. 1a), encompassing various outcome measures reflecting score changes, such as the SCORing Atopic Dermatitis (SCORAD) index, Eczema Area and Severity Index (EASI), Dermatology Life Quality Index (DLQI), Numeric Rating Scale (NRS), Transepidermal Water Loss (TEWL), capacitance-based skin hydration, Investigator's Global Assessment (IGA), and Body Surface Area (BSA). The network graphs illustrating the comparative relationships among these indicators are presented in Fig. 1b, where nodes represent competing indicators and edges denote RCTs evaluating pairs of indicators. The risk of bias assessment for these trials was conducted using the Cochrane Risk of Bias 2.0 tool, indicating that 42.5% of the included trials were classified as having a low risk of bias (Fig. 1c) [5].

**Fig. 1:**
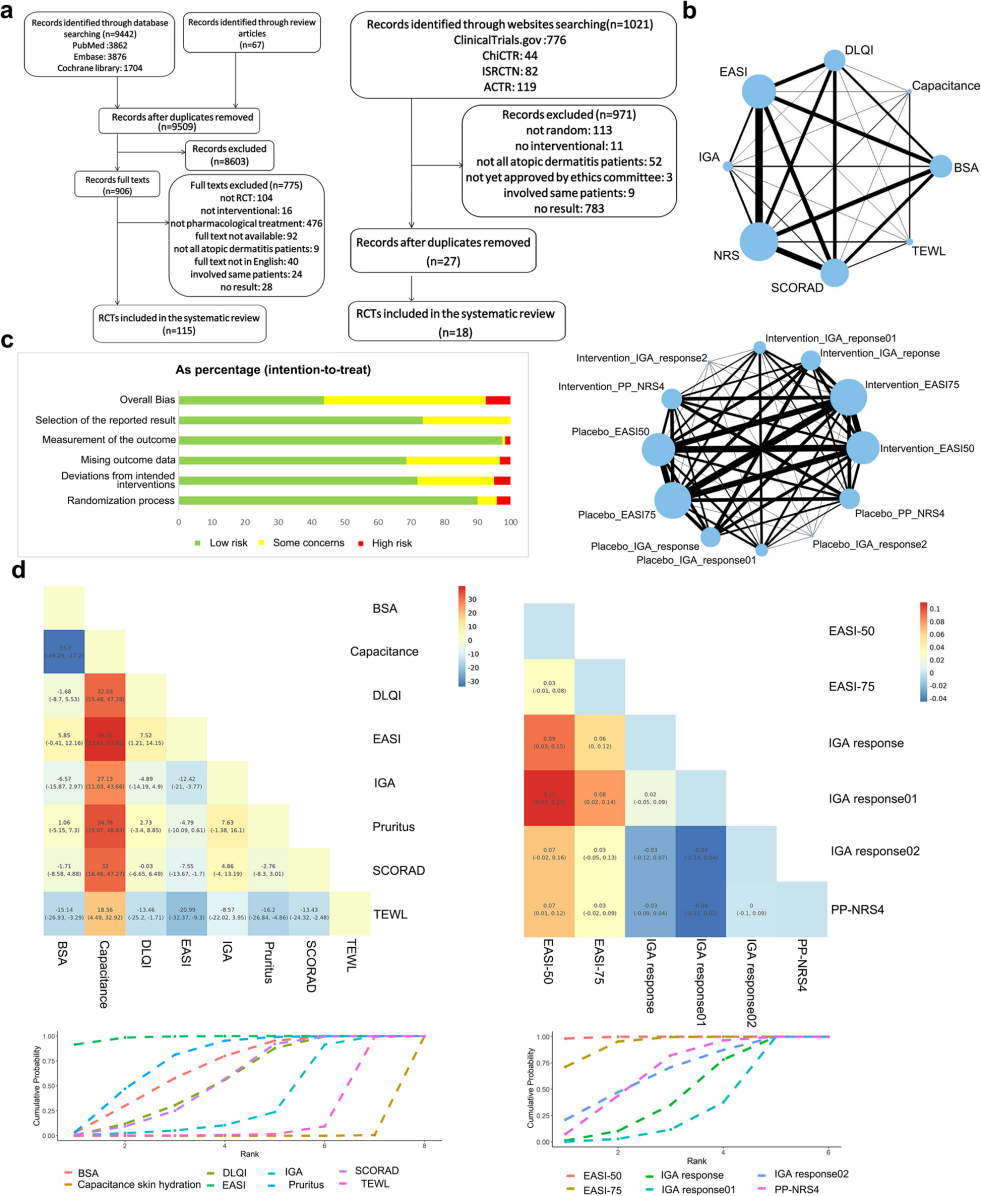
Indicator preference for reporting score changes and response rate in RCTs with pharmacological intervention for AD. **a** Literature search and selection from published articles and clinical trials registries. **b** Network of eligible comparisons for efficacy evaluation indicators reporting score change (up) and response rate (down). The size of the nodes (blue circles) corresponds to the number of trials. Comparisons are linked with a line, the thickness of which corresponds to the number of trials that assessed the comparison. **c** Risk of bias summary graph: review authors’ judgements (Low, Some concerns, and High) for each risk of bias item shown as percentages across all included studies. **d** Effectiveness estimates with 95% confidence intervals for indicators reporting score change (left) and response rate (right) in RCTs with pharmacological intervention for AD. Ranking of indicators based on their ability to report score changes and response rate. Indicators that achieve rank 1 earlier demonstrate a stronger capacity to distinguish intervention efficacy

According to Bayesian model estimation, considering the influence of different intervention types, various routes of drug administration, subjects' age, and the severity of AD, EASI was the best indicator with significant greater intervention- and control-treatment score difference compared to other indicators, which meant for the same participants, EASI was more effective in detecting the impact of pharmacological interventions compared to other indicators (Fig. 1d left). Instead, capacitance was shown to be the worst indicator, its intervention- and control-treatment score had no significant difference (Fig. 1d left), which meant it was insufficient in highlighting the differences between pharmacological interventions and controls. Besides, TEWL also showed to be the less effectiveness indicator, which had lower intervention- and control-treatment score difference compared to DLQI, BSA, NRS, SCORAD, and IGA (Fig. 1d left). Other than that, there was no clear distinction between other indicators (Fig. 1d left).

The preference for indicators reporting score change was also evident when evaluating different disease severities, application types, intervention types, and age stratified patient groups. EASI demonstrated higher sensitivity in assessing treatment outcomes among severe AD patients, but its sensitivity decreased for mild to moderate AD participants. EASI outperformed DLQI, TEWL, and capacitance, while being comparable to BSA and NRS. In contrast, NRS showed greater sensitivity for evaluating treatment effects in mild to moderate AD patients, but its sensitivity decreased in moderate to severe AD cases. DLQI and TEWL had limited discrimination ability, performing significantly worse than EASI and NRS for mild to moderate AD participants. Capacitance remained the least effective indicator across all comparisons. Among moderate to severe AD patients, despite less discrepancy in mean relative score changes, EASI continued to be the strongest indicator of AD, showing significant score differences compared to IGA and BSA.

EASI was also the most powerful indicator for evaluating topical pharmacological interventions, with a significantly higher relative score change percentage after treatment compared to other indicators, except IGA and NRS. TEWL and capacitance remained less effective, consistent with the overall assessment. For systemic drug interventions, IGA and NRS showed reduced evaluation capabilities, with IGA having the lowest sensitivity. EASI continued to demonstrate its superiority in systemic drug intervention RCTs, outperforming IGA and SCORAD, and showing no dominant advantage over NRS. Meanwhile, the evaluation sensitivity of BSA and DLQI improved, placing them just below EASI.

For small molecule treatments, EASI was the most effective indicator, outperforming BSA, capacitance, IGA, SCORAD, and TEWL, while capacitance remained the least effective. Other indicators showed no significant differences in evaluating the efficacy of small molecules. When assessing antibody treatment efficacy, the performance of EASI diminished, although it still maintained certain advantages over other indicators. For non-biologic agents, BSA emerged as the most robust indicator, followed by NRS and EASI.

The preference for response rate indicators was evaluated using a Bayesian hierarchical model. EASI-50 demonstrated the highest response rate in intervention groups compared to control groups, outperforming EASI-75, IGA response, IGA response01, and PP-NRS4 (Fig. 1d right). This suggests that EASI-50 is more effective in identifying the efficacy of pharmacological interventions among indicators reporting response rates (Fig. 1d right). While other indicators reporting response rate had no clear distinction mutually (Fig. 1d right).

Indicators reporting response rate were influenced by disease severity, application type, intervention type, and age stratification. For mild to moderate AD patients, no significant differences were observed between response rate indicators. However, for moderate to severe AD patients, EASI-50 outperformed IGA response, IGA response01, and PP-NRS4, while EASI-75 ranked second, showing superiority over IGA response. In severe AD cases, EASI-50 and EASI-75 were superior to IGA response01, with no significant differences among other indicators.

For topical treatments, EASI-50 was the most effective indicator, significantly outperforming IGA response and IGA response01. IGA response was considered the least effective, despite lacking statistical significance. For systemic treatments, EASI-50 demonstrated clear superiority over IGA response01 and PP-NRS4, with EASI-75 also outperforming IGA response01.

When evaluating small molecule treatments, EASI-50 showed better performance compared to other indicators, though no statistical differences were found. For antibody treatments, EASI-50 again demonstrated strong efficacy, outperforming both IGA response and IGA response01. In non-biologic treatments, no significant differences in response rate indicators were observed.

In adult AD patients, EASI-50 was preferred over IGA response and IGA response01 for measuring treatment efficacy, with EASI-75 also showing better efficacy than IGA response. Both EASI-50 and EASI-75 showed good efficacy discrimination in pediatric AD patients. EASI-50 was the best indicator for reporting response rate and showed significantly better results than EASI-75, IGA response, and IGA response01. Moreover, selecting EASI-75 yielded more significant results than IGA response and IGA response01.

The limitation of this study lies in its reliance on retrospective data from registered clinical trials, which may introduce selection bias and limit the generalizability of the findings to broader AD populations. Despite these limitations, the findings provide a valuable foundation for selecting and standardizing outcome indicators in AD clinical trials, contributing to improved comparability of trial evidence and enhancing evidence-based treatment decisions. Future studies should focus on validating these findings prospectively in larger, diverse populations, as well as exploring novel indicators and advanced modeling approaches to further optimize efficacy evaluation in AD.

In conclusion, our study offers significant insights into comparing trial evidence and establishing primary outcomes in clinical research on AD. To effectively assess and highlight the therapeutic benefits of pharmacological interventions, we advocate for the adoption of EASI scores and EASI-50, given their superior sensitivity, as primary outcomes in AD RCTs. Furthermore, comprehensive analyses incorporating other indicators are deemed essential. In trials solely reliant on EASI-50 or NRS evaluations, careful attention is warranted during outcome interpretation to prevent overestimation of treatment efficacy.

## Supplementary Information

Supplementary Material 1.

## Data Availability

All data supporting the findings of this study are available within the paper and its Supplementary Information.
