# Supplementary material for: Evaluation of outcome indicators in trials for atopic dermatitis
Source: Mol Biomed. 2025 May 26;6:33. doi: 10.1186/s43556-025-00273-8 (PMC12106173; doi:10.1186/s43556-025-00273-8)
Supplement: Supplementary file 1 — Supplementary Material 1. [file 43556_2025_273_MOESM1_ESM.docx]

SUPPLEMENTARY MATERIAL

Evaluation of outcome indicators in trials for atopic dermatitis

Jingru Tian, M.D.^1,2#^, Liqing Shi,M.D. ^1,2#^, Shuntong Kang, M.D.^3^, Dingyao Zhang, B.S.^4^, Yaqing Huang M.S.^5^, Ming Zhao, M.D., Ph.D.^1,2,3^*, Xu Yao, M.D., Ph.D.^1^*, and Qianjin Lu, M.D., Ph.D.^1,2^*

^1^Hospital for Skin Diseases, Institute of Dermatology, Chinese Academy of Medical Sciences and Peking Union Medical College, Nanjing, China.

^2^Key Laboratory of Basic and Translational Research on Immune-Mediated Skin Diseases, Chinese Academy of Medical Sciences, Nanjing, China

^3^Department of Dermatology, Hunan Key Laboratory of Medical Epigenomics, The Second Xiangya Hospital, Central South University, Changsha, Hunan, China.

^4^Graduate Program in Biological and Biomedical Sciences, Yale University, New Haven, Connecticut 06510, USA

^5^Department of Pathology, Yale University, New Haven, CT, 06520, USA

# These authors contribute equally to the work.

* **Correspondence authors**:

Qianjin Lu, 12 Jiangwangmiao Street, Xuanwu, Nanjing, Jiangsu, China 210042.

Telephone: 025-85478999; Fax: 025-85414477;

Email: [qianlu5860@pumcderm.cams.cn](mailto:qianlu5860@pumcderm.cams.cn)

Xu Yao, 12 Jiangwangmiao Street, Xuanwu, Nanjing, Jiangsu, China 210042.

Telephone: 025-85478999; Fax: 025-85414477;

Email: [dryao_xu@126.com](mailto:dryao_xu@126.com)

Ming Zhao, 12 Jiangwangmiao Street, Xuanwu, Nanjing, Jiangsu, China 210042.

Telephone: 025-85478999; Fax: 025-85414477;

Email: [zhaoming301@pumcderm.cams.cn](mailto:zhaoming301@pumcderm.cams.cn)

Materials and Methods

| 1. **Search strategy**   Two investigators (JR.T and LQ.S) searched published articles and clinical trial registry records, and appraised studies on eligibility, and extracted data independently. Discrepancies were discussed and agreed by consensus.  The search for RCTs included published articles from peer-reviewed English-language journals and registered trials in clinical trials registries, both up to April 09, 2024 and without start date restriction. The published articles were searched in literature databases including the PubMed, EMBASE, and Cochrane Library Central Register of Controlled Trials (CENTRAL). The MeSH and keyword search terms associated with atopic dermatitis were used in each database. In order not to miss out on potentially useful articles, references cited in relevant reviews were also searched manually. RCTs published in Chinese medical journals were also included.  Records of registered RCTs were collected from 4 publicly available web-based clinical trials registries, including the ClinicalTrials.gov of the US National Library of Medicine, the International Standard Randomised Controlled Trial Number Register (ISRCTN), the Australian and New Zealand Clinical Trials Registry (ANZCTR), and the Chinese Clinical Trial Register. The keyword search term “atopic dermatitis” or “eczema” was entered combined with other specific filtering options in advanced search function for ‘Country’, ‘Study type’, and ‘Current status’ et al. in searching for eligible RCTs. |
| --- |
| 1. **Study selection**   Initially, published articles were evaluated based on their titles or abstracts by two independent investigators according to predefined inclusion and exclusion criteria. In cases where discrepancies arose, the investigators first revisited the predefined criteria to discuss the specific points of disagreement. If consensus could not be reached through discussion, a third independent investigator, with expertise in atopic dermatitis evaluation studies, was consulted to adjudicate the final decision. All decisions were documented to ensure transparency and reproducibility. Articles showing potential relevance were then further scrutinized as complete reports against predefined selection criteria. Potentially relevant articles were thoroughly evaluated as complete reports based on predefined selection criteria. Eligible studies included both published works and registered trial records that involved patients with AD who were randomly allocated to distinct intervention groups. The exclusion criteria included: 1) studies involving non-human subjects; 2) studies lacking patients diagnosed with AD or with ambiguous diagnostic methods; 3) observational research studies without interventions related to AD treatment; 4) studies not incorporating a randomization process; and 5) studies lacking approval from an ethics committee.  Furthermore, published articles meeting any of the following criteria were excluded: 1) not written in English; 2) lacking full-text availability (e.g., abstracts, conference proceedings) or not reporting original studies (e.g., narrative reviews, meta-analyses, editorials, commentaries, protocols, guidelines, or perspectives); or 3) duplicate reports. The search strategy for literature retrieval from published articles and records from clinical trial registries is detailed in Appendix 2. |
| 1. **Data extraction**   Two investigators independently extracted information on characteristics of each included study, including general information (author, publication year, registration ID, year of start, domestic or multinational, single- or multi-center, affiliations of primary investigators), participant characteristics (subject type, number of participants, loss to follow-up), study intervention (measures of intervention or control, duration, blinding), and primary outcomes. Some information of participant characteristics was not available for multinational trials because they did not provide information separately for participants in individual countries. Extracted data from published articles and records from clinical trials registries were entered separately into two piloted spreadsheets, and then combined together matched by the registration ID or other information if the registration ID was unavailable. For studies with data available from both sources, data from published articles were used. Potential duplicate registry entries were searched for by matching on important trial characteristics including year of start, affiliation of primary investigator, subject category, number of participants, interventions, and primary outcome. Published trials which did not include a trial registration ID was considered not registered. |
| 1. **Study categorization**   We included RCTs conducted in subjects with atopic dermatitis only (including atopic dermatitis; atopic eczema; nummular eczema; and prurigo nodularis), and atopic dermatitis with comorbidities (including food, aeroallergen, yeast, or dust mite allergy; atopic keratoconjunctivitis; gastrointestinal disorders; sleep disturbance; and infection). The following are the characteristics of the included RCTs on atopic dermatitis conducted in China.  **Table 1. Characteristics of included RCTs of atopic dermatitis conducted in China**   \| **Categories** \| **No (%)** \| \| --- \| --- \| \| **Data repository** \| \| \| Published articles \| 117 (88.0) \| \| Clinical trials registries \| 16 (12.0) \| \| **Center** \| \| \| Single center \| 59 (44.4) \| \| Multiple centers \| 71 (53.4) \| \| Not mentioned \| 3 (2.3) \| \| **Year of start** \|  \| \| Before 2014 \| 29 (21.8) \| \| 2014-2017 \| 49 (36.8) \| \| 2017-2024 \| 36 (27.1) \| \| Not available \| 19 (14.3) \| \| **No. of participants** \|  \| \| <50 \| 27 (20.3) \| \| 50-99 \| 37 (27.8) \| \| 100-199 \| 34(25.6) \| \| ≥200 \| 14 (10.5) \| \| Not mentioned \| 16 (12.0) \| \| **Disease severity** \|  \| \| Mild \| 5 (3.8) \| \| Mild to Moderate \| 30 (22.6) \| \| Moderate \| 4 (3.0) \| \| Moderate to Severe \| 68 (51.1) \| \| Severe \| 6 (4.5) \| \| All \| 10 (7.5) \| \| Not Mentioned \| 10 (7.5) \| \| **Blinding** \|  \| \| Single blind \| 4 (3.0) \| \| Double blind \| 115 (86.5) \| \| Open label \| 3 (2.3) \| \| Others \| 10 (7.5) \| \| Others (Quadruple) \| 7 (5.3) \| \| Others (Triple) \| 3 (2.3) \| \| Not mentioned \| 1 (0.8) \| \| **Intervention duration (months)** \|  \| \| <1 \| 10 (7.5) \| \| 1-2.9 \| 46 (34.6) \| \| 3-5.9 \| 65 (48.9) \| \| 6-8.9 \| 6 (4.5) \| \| ≥9 \| 6 (4.5) \| \| **Type intervention** \|  \| \| Small molecules \| 47 (35.3) \| \| Antibodies \| 42 (31.6) \| \| Non-biologics \| 44 (33.1) \| \| **Dosing method** \|  \| \| Topical \| 77 (57.9) \| \| Systemic \| 56 (42.1) \| |
| **Statistical methods for indicator preference calculation**  The model fitted by a Bayesian hierarchical linear mixed model. In hierarchical model, the effectiveness of an intervention was estimated based on study data both from the same intervention and from other interventions in the same type of Interventions.  The model applied binomial family, and log-transformation was used to transform effectiveness to a linear response variable. The statistical was implemented by brms package in R (version 4.0.5). This package is based on Stan and will estimate posterior distribution by Hamiltonian Markov Chain Monte Carlo method. Four chains were used, and the warmup number and iteration number are both 4000. Besides interventions, we have set another three predictor variables with fixed effects. The four fixed variables are indicator type, severity of the disease, whether the intervention is topical, and age of the patients.  Additionally, the model used a student_t(3, 0, 2.5) prior for the intercept. We reported the effectiveness estimate with 95% uncertainty intervals. Finally, we also assessed the models in total, the fit of each model was assessed by effective sample size, autocorrelation, and trace plots. Please contact Dingyao Zhang for the code of model estimation.  For continue model:  model <- brm(mean \| se(sd, sigma = TRUE) ~ 1 + (1 \| index) + (1 \| Type_intervention/Interventions) + (1 \| Severity) + (1 \| Topical) + (1 \| Age), data = datause, thin = 10, chains = 4, iter = 8000, cores = 4, control = list(adapt_delta = 0.999, max_treedepth=20))  For discrete model:  model <- brm(mean \| se(sd, sigma = TRUE) ~ 1 + (1 \| index) + (1 \| Type_intervention/Interventions) + (1 \| Severity) + (1 \| Topical) + (1 \| Age), data = datause, thin = 10, chains = 4, iter = 8000, cores = 4, control = list(adapt_delta = 0.9995, max_treedepth=20))  Building on previous data, we provide recommendations for selecting the primary outcome in RCTs for AD, as presented in the table below.  **Table 2. Recommendations for the selection of primary outcome of RCTs for AD**   \| **Items** \| **Suggested Indicators** \| \| **Not suggested Indicators** \| \| \| --- \| --- \| --- \| --- \| --- \| \|  \| **Score change** \| **Response rate** \| **Score change** \| **Response rate** \| \| **Overall** \| EASI \| EASI-50  EASI-75 \| Capacitance  TEWL  SCORAD \| IGA response0/1  PP-NRS4  IGA response \| \| **AD baseline severity** \|  \|  \|  \|  \| \| Mild to moderate \| EASI  BSA  NRS \| EASI-50  PP-NRS4 \| Capacitance  TEWL \| - \| \| Moderate to severe \| EASI \| EASI-50  EASI-75 \| - \| IGA response0/1  PP-NRS4  IGA response \| \| **Age of participants** \|  \|  \|  \|  \| \| Adult \| EASI \| EASI-50 \| Capacitance  TEWL \| IGA response0/1  IGA response \| \| Underage \| EASI  NRS \| EASI-50  EASI-75 \| - \| IGA response  IGA response0/1 \| \| **Application method** \|  \|  \|  \|  \| \| Topical \| EASI  NRS \| EASI-50 \| Capacitance  DLQI  TEWL \| IGA response0/1  IGA response \| \| Systemic \| EASI  DLQI  BSA \| EASI-50  EASI-75 \| - \| PP-NRS4 \| \| **Antibodies or small molecules** \| \| \| \| \| \| Antibodies \| EASI \| EASI-50 \| - \| IGA response0/1  IGA response \| \| Small molecules \| EASI  NRS  DLQI \| EASI-50  EASI-75 \| Capacitance \| IGA response  PP-NRS4 \| \| Non-biologics \| EASI \|  \| - \| - \| \| **Minor efficacy** \| NRS \| EASI-50 \| - \| - \|   BSA: and body surface area affected, DLQI: Dermatology Life Quality Index, EASI: Eczema Area and Severity Index, EASI-50: 50% decrease in EASI, EASI-75: 75% decrease in EASI, IGA: investigator global assessment, IGA response: clear or almost clear (grade 0 or 1) and at least a two-grade improvement from baseline, IGA response0/1: clear or almost clear (grade 0 or 1), NRS: pruritus numerical rating scale, PP-NRS4: ≥4-point improvement from baseline using Peak Pruritus Numerical Rating Scale, SCORAD: SCORing atopic dermatitis, TEWL: transepidermal water loss. |
